# How to Treat Breast Cancer After Childhood Cancer: Management of Oncologic and Cardiovascular Concerns

**DOI:** 10.1016/j.jaccao.2021.12.003

**Published:** 2022-03-15

**Authors:** Anne H. Blaes, Suma Konety, Jane Hui, Kathryn Dusenbery, Jianling Yuan, Susan Dent, Kevin C. Oeffinger, Lucie M. Turcotte

**Affiliations:** a Division of Hematology/Oncology, University of Minnesota, Minneapolis, Minnesota, USA; b University of Minnesota, Masonic Cancer Center, Minneapolis, Minnesota, USA; c Division of Cardiology, University of Minnesota, Minneapolis, Minnesota, USA; d Division of Surgical Oncology, University of Minnesota, Minneapolis, Minnesota, USA; e Department of Radiation Oncology, University of Minnesota, Minneapolis, Minnesota, USA; f Duke University, Duke Cancer Institute, Raleigh, North Carolina, USA; g Division of Pediatric Oncology, University of Minnesota, Minneapolis, Minnesota, USA

**Keywords:** cancer survivorship, coronary artery disease, heart failure

## Abstract

• Childhood cancer survivors are at higher risk for the development of breast cancer necessitating early breast cancer screening, often with both breast MRI and mammography.
• Risk-stratify breast cancer treatment, taking into account prior radiation fields, surgical procedures, use of anthracyclines, and current comorbidities is essential.
• Aggressive management of CV risk factors in collaboration with cardiologists, oncologists, primary care providers, and allied health care providers is needed to provide the best cancer treatment while optimizing CV health.

Survival following a diagnosis of childhood cancer has improved dramatically, with 5-year survival exceeding 85%. Unfortunately, long-term survival comes at a cost to the survivor in the form of long-term health consequences. Subsequent malignancies, the most common of which is breast cancer among women, are associated with significant morbidity and mortality.^1^^,^^2^ Therapeutic radiation to the chest and use of anthracycline chemotherapy are significant risk factors for the development of cardiovascular disease (CVD). Among survivors of childhood cancer who received chest radiotherapy for their primary malignancies, the cumulative breast cancer incidence by 50 years of age approaches 30%, similar to that of women who carry *BRCA1* mutations.^1^ Importantly, Moskowitz et al^2^ reported that by 15 years after a breast cancer diagnosis, overall survival was only 50%. Half of the deaths were related to the breast cancer, while the other deaths were from CVD or other subsequent malignancies. Thus, the diagnosis of breast cancer may provide an opportunity for prevention and early intervention. Furthermore, although the risk factors associated with developing breast cancer after childhood cancer are well understood, there are fewer data on appropriate treatment options for those diagnosed with breast cancer.

This primer provides clinical cases focusing on the challenges associated with treating breast cancer after surviving childhood cancer.

## Case 1

A 50-year-old woman with a history of hypertension and diabetes presents with a T2N0M0 triple-negative (estrogen receptor [ER] negative, progesterone receptor [PR] negative, human epidermal growth factor receptor 2 [HER2] negative) breast cancer of the left breast. She was treated at 4 years of age for Wilms’ tumor with right nephrectomy, flank and bilateral whole-lung radiation, and multiagent nonanthracycline chemotherapy. Body mass index is >30 kg/m^2^ and blood pressure is 145/80 mm Hg. Medications include carvedilol, metformin, aspirin 81 mg/d. Treatment with 4 cycles of neoadjuvant doxorubicin (60 mg/m^2^) and cyclophosphamide (600 mg/m^2^) given intravenously every 2 weeks followed by paclitaxel (80 mg/m^2^) intravenously for 12 weeks is prescribed. Baseline 2-dimensional echocardiography reveals a left ventricular ejection fraction (LVEF) of 50%. Baseline electrocardiography shows normal sinus rhythm with mild left ventricular (LV) hypertrophy.

This patient is at high risk for incident breast cancer and cardiovascular (CV) complications from her prior radiation. Current guidelines recommend screening mammography and breast magnetic resonance imaging annually beginning 8 years after radiation or at the age of 25 years, whichever comes last.^3^ Dual-modality breast cancer screening has been shown to improve overall survival and breast cancer mortality. Tamoxifen may be considered as a preventive approach.^4^

Triple-negative breast cancer is a biologically aggressive cancer in which combination chemotherapy with an anthracycline and taxane with or without carboplatin and immunotherapy is recommended and is generally well tolerated.^5^ Adjuvant capecitabine may be given for 6 months if pathologic complete response (pCR) is not achieved. Given this patient’s prior radiation exposure and current need for anthracyclines, it is important to consider her CVD risk (Table 1).

Cardiology and oncology professional society guidelines recommend baseline CV risk assessment for all patients with cancer prescribed potentially cardiotoxic therapy, but guidance is limited on how to define CV risk for individual patients. Risk is a continuous variable, and multiple factors may coexist. Weighing both patient comorbidities and treatment-related CV risk factors should be considered, and expert-consensus based calculators including those developed by the Cardio-Oncology Study Group from the Heart Failure Association of the European Society of Cardiology could be considered.^6^ For this patient receiving anthracyclines, several risk factors emerge: borderline LVEF (50%-54%), medium risk (2 points); hypertension, medium risk (1 point); diabetes, medium risk (1 point) and BMI >30 kg/m^2^, medium risk (1 point); the total risk score is 5 points. Patients with scores ≥ 5 points are deemed at high risk, and referral for cardio-oncologic assessment is recommended. Patients at low to medium risk can be followed closely, with less vigorous cardiac monitoring, per current guidelines. Although validation studies are needed, this is an important contribution to personalizing CV risk assessment in patients with cancer.

Recognizing the extremely high risk for an asynchronous breast cancer and the high mortality after breast cancer diagnosis (50% by 15 years after diagnosis), and given the wide and bilateral field of radiation for Wilms’ tumor, a bilateral mastectomy (prophylactic right) should be recommended. Breast conservation, however, is not entirely contraindicated. A dose of 40 to 42.5 Gy over 15 to 16 fractions to the whole breast is generally considered safe following remote treatment with 12 Gy to the lung. Cardiac-sparing techniques such as deep-inspiration breath hold or prone positioning should be used. The risk for secondary malignancies and radiation-induced pulmonary and cardiotoxicities should be considered.

Given the accelerated aging of childhood cancer survivors and the impact of cancer therapy on exercise tolerance, referral to a cancer rehabilitation program is highly recommended.^7^ In addition, lifestyle modification with exercise and CV risk factor modifications can reduce comorbid complications and may aid in improving breast cancer outcomes.

## Case 2

A 39-year-old postmenopausal woman with a history of hypertension presents with 2-cm, node-negative, ER+/HER2− left-sided breast cancer. The Oncotype DX score was 15. She was treated at 15 years of age for sarcoma with “red chemotherapy and multiple other drugs.” After reviewing records, it was found that she received 400 mg/m^2^ anthracycline and developed premature menopause with her prior therapy. Genetics referral is provided.

The standard surgical approach for early-stage hormone-positive breast cancer is treatment with lumpectomy, sentinel lymph node biopsy, and adjuvant radiation therapy. Mastectomy with sentinel lymph node biopsy results in equivalent breast cancer recurrence outcomes and no improvement in overall survival.^8^ In the TAILORx (Trial Assigning Individualized Options for Treatment) study, there was no benefit to adjuvant chemotherapy in node-negative postmenopausal woman with a low Oncotype Dx score (<25).^9^ Adjuvant endocrine therapy with an aromatase inhibitor (AI) is recommended for 5 years, with consideration for 10 years.

Cancer survivors often cannot recall the names of chemotherapies they received, and obtaining records from decades prior is difficult. On the basis of CV risk calculators developed and validated by the Childhood Cancer Survivor Study, this patient’s risk for heart failure by 50 years of age is 9.7%, a 31-fold higher risk compared with a sibling control without cancer.^10^ Aggressive CV risk factor management, including blood pressure, lipid, and glucose control, can improve CV outcomes. Echocardiography every 2 years is recommended by the Children’s Oncology Group in this scenario.

Adjuvant endocrine therapy with tamoxifen or an AI reduces the risk for recurrence by approximately one-third. In postmenopausal women, an AI would be recommended given superiority in reduction of risk for breast cancer recurrence. Large clinical studies have reported higher rates of hypertension, hypercholesterolemia, and ischemic CV disease in postmenopausal breast cancer survivors receiving AIs. This patient’s prior anthracycline exposure, premature menopause, and use of AIs necessitate aggressive CV risk factor management with risk-reducing medications and exercise under the direction of a cardio-oncologist or primary care provider.

## Case 3

A 40-year-old woman with a history of Hodgkin lymphoma treated with ABVD chemotherapy (doxorubicin 300 mg/m^2^, bleomycin, vinblastine, and dacarbazine) and mantle radiation at 19 years of age presents with a stage IIB (T2N1) lymph node–positive ER−/HER2+ breast cancer. Neoadjuvant chemotherapy with a non-anthracycline-based regimen with trastuzumab and pertuzumab (HP) is recommended.

Stage IIB HER2-positive breast cancer is treated with neoadjuvant chemotherapy using a trastuzumab-based regimen. Although trastuzumab and taxane alone can be considered for stage I and early stage II disease with excellent disease-free and overall survival, for stage IIB disease, dual HER2 blockade using HP in combination with chemotherapy (docetaxel, carboplatin, trastuzumab, and pertuzumab) would be considered optimal.^11^ Anthracycline-based regimens should be avoided.

This patient is at increased risk for cancer therapy–related cardiac dysfunction, defined as a decrease in LVEF of >10% to a value less than the lower limit of normal. A baseline comprehensive CV assessment including 3-dimensional echocardiography with global longitudinal strain and consideration for prophylactic cardioprotective medications is recommended prior to treatment.

As previously discussed, bilateral mastectomy would be recommended given the patient’s elevated risk for additional future de novo breast cancers. Adjuvant radiation therapy may still need to be considered given the elevated risk for locoregional recurrence. Although retrospective data are reassuring, reporting a low risk for grade 3 toxicity,^12^ patients should be counseled about the increased risk for skin fibrosis, chest wall necrosis, rib fracture, and brachial plexopathy. Discussion among the patient, radiation oncologist, and medical oncologist is essential. Proton therapy may confer an improved dosimetry compared with conventional photon therapy in the setting of reirradiation. Proactive engagement of cancer rehabilitation and lymphedema are recommended.

The patient completes docetaxel, carboplatin, trastuzumab, and pertuzumab and undergoes surgery with a full axillary node dissection. She has pCR. Cancer treatment with 1 year of postoperative HP is recommended. Follow-up echocardiography after the first cycle of HP demonstrates a decline in LVEF to 44%.

In the adjuvant setting, trastuzumab with or without pertuzumab is recommended every 3 weeks to complete 12 months of therapy. Trastuzumab emtansine can be offered in the adjuvant setting for those who do not achieve pCR^8^; there appears to be minimal cardiac risk. Echocardiography every 3 months, with strain assessment when available, should be continued for cardiac surveillance during HER2-targeted therapy.

In an individual with prior exposure to anthracyclines and chest radiation, this decline in LVEF is suggestive of cancer therapy–related cardiac dysfunction related to HER2 therapy. Other causes, including metabolic and ischemic etiologies (particularly given prior chest radiation), must be excluded.

The SAFE-HeaRt (Cardiac Safety Study in Patients With HER2^+^ Breast Cancer) trial, which enrolled HER2-positive patients with asymptomatic LV dysfunction (LVEF 40%-49%) who were candidates for trastuzumab, pertuzumab, or trastuzumab emtansine,^13^ demonstrated that it is safe to use HER2-targeted therapies in select asymptomatic patients with LV dysfunction. These patients should receive appropriate medications (beta-blockers and angiotensin-converting enzyme inhibitors) and close cardiac monitoring with cardiology.^14^ This is important given the major advance that HER2-targeted therapies represent for these challenging patients.

Individuals who develop metastatic HER2-positive breast cancer will be exposed to sequential HER2-based therapies. For these patients, there is no clear guidance on the frequency of cardiac monitoring. If LVEF is stable for 12 months, the patient remains asymptomatic, and natriuretic peptide biomarkers remain stable, it may be reasonable to consider reducing the interval of echocardiography frequency to 6 months or more. This modification should be made on an individual basis, weighing prior cardiotoxic exposures and comorbid risk factors.

## Conclusions

Care for childhood cancer survivors who develop breast cancer requires careful consideration of prior cancer treatments, as well as comorbidities that frequently develop in the decades following childhood cancer diagnosis. Aggressive management of CV risk factors in collaboration with cardiologists, oncologists, primary care providers, and allied health care providers is needed to provide the best cancer treatment while optimizing CV health.

**Table 1:** Approach to Childhood Cancer Survivors With New Breast Cancer

1. Obtain history and treatment on prior childhood cancer treated, recognizing that not all information may be obtainable, focusing on fields of radiation, use of anthracyclines, age at treatment as a child, and current menopausal status of patient.
2. Recognize that all patients need optimization of CV risk factors with the potential for risk-reducing medications and lifestyle management for a healthy weight, diet, exercise, tobacco cessation.
3. Workup for new breast cancer diagnosis as per standard NCCN guidelines, including referral to cancer genetics.
4. Risk stratify breast cancer treatment, taking into account prior radiation fields, surgical procedures, use of anthracyclines, and current comorbidities.
5. Obtain baseline cardiac imaging, electrocardiography, and consideration for biomarkers (including natriuretic peptides).
6. Consider referral to cardio-oncologist to discuss risk-reducing strategies, cardioprotective medications, and potential ongoing use of blood biomarkers (natriuretic peptides).
7. Incorporate physical therapy and cancer rehabilitation into breast cancer treatment to minimize long-term complications with lymphedema and reduced mobility, particularly for those with prior radiation in the treatment of their childhood cancer.
8. Emphasize the importance of cardiac risk-reducing strategies in all patients with close collaboration of primary care and/or cardio-oncology.

CV = cardiovascular; NCCN = National Comprehensive Cancer Network.

## Funding Support and Author Disclosures

This work was supported by NIH grant K08 CA234232 to Dr Turcotte. All other authors have reported that they have no relationships relevant to the contents of this paper to disclose.
